# 

**Published:** 1933

**Authors:** 


### The Medical Library of the University of Bristol,

WITH WHICH IS INCORPORATED

**The Library of the Bristol Medico-Chirurgical Society.**

---

*The following donations have been received since the publication of the list in June,* 1933.

November, 1933.

| | | | |
|---|---|---|---|
| American Laryngological Association (1) | .. | 1 | volume. |
| British Hanovia Quartz Lamp Co. Ltd. (2) | .. | 1 | ,, |
| Carnegie Institution of Washington (3) | .. | 1 | ,, |
| Granada University (4) .. | .. .. .. | 1 | ,, |
| Professor E. W. Hey Groves (5) | .. .. | 1 | ,, |
| Dr. Jonas (6) | .. .. .. .. .. | 1 | ,, |
| Otago University (7) | .. .. .. .. | 1 | ,, |
| Purcell Research Memorial (8) | .. .. | 1 | ,, |
| Rockefeller Foundation (9) | .. .. .. | 1 | ,, |
| Royal College of Surgeons of England (10) | .. | 1 | ,, |
| Professor R. S. S. Statham (11) | .. .. | 1 | ,, |
| Wilmer Institution of the Johns Hopkins University (12) | .. .. .. .. | 1 | ,, |
| John Wright & Sons Ltd. (13) | .. .. | 1 | ,, |

Unbound periodicals have also been received from Professor E. W. Hey Groves.

### THE ONE HUNDRED AND FORTY-FIFTH LIST OF BOOKS.

The figures in round brackets refer to the figures after the names of the donors. The books to which no such figures are attached have either been bought from the Library Fund or received through the *Journal.*

**Abrahams, A.** .. .. *Diseases and Disorders of the Digestive Organs* 1932

*Actinotherapy Technique* .. .. .. .. .. .. .. .. .. .. .. (2) [1933]

**Beaumont, G. E.** .. *Medicine : Essentials for Practitioners and Students* .. .. .. .. .. .. .. .. .. 1932

X

| | | |
|---|---|---|
| **Berry, R. J. A.** | *Stoke Park Studies, Series* 1. *Mental Deficiency* | 1933 |
| **Bickerton, J. M., and Savin, L. H.** | *Clinical Ophthalmology* | 1933 |
| **Blacklock, D. D.** | *Empire Problem* | 1932 |
| **Bostock, J.** | *Neural Energy Constant* | 1931 |
| **Brain, W. R.** | *Diseases of the Nervous System* | 1933 |
| **Buchanan, A.** | *Midwifery Mechanics* (11) | N.D. |
| **Cameron, A. J. D.** | *Some Thoughts on Asthma* | 1933 |
| **Charles, E.** | *Practice of Birth Control* | 1932 |
| **Clay, H. H.** | *Sanitary Inspector's Handbook* | 1933 |
| **Combe, A.** | *Principles of Physiology* 5th Ed. | 1836 |
| **Core, D.** | *Examination of the Central Nervous System* | 1928 |
| **Crocket, J.** | *Physical and Radiological Examination of the Lungs* 2nd Ed. | 1931 |
| **Devine, H.** | *Recent Advances in Psychiatry* | 1929 |
| **Douthwaite, A. H.** | *Treatment of Rheumatoid Arthritis and Sciatica* 2nd Ed. | 1933 |
| **Ellis, Havelock** | *Psychology of Sex* | 1933 |
| **Ellman, P.** | *Chest Disease in General Practice* | 1932 |
| **Fanning, J.** | *Outline of Medicine for Nurses* | 1933 |
| **Fuller, E.** | *On the Management of a Birth Control Centre.* 2nd Ed. | [1931] |
| **Gallichan, W. M.** | *Critical Age of Women* | 1932 |
| **Goodwin, F.** | *Account of the Neutral Saline Waters at Hampstead* | 1804 |
| **Gordon, R. G.** | *Neurotic Personality* | 1927 |
| **Gordon, R. S., and Brown, M. F.** | *Paralysis in Children* | 1933 |
| **Gourmont, R. de** | *Natural Philosophy of Love* | 1926 |
| **Groves, E. W. H.** | *Surgical Operations* | 1933 |
| **Groves, E. W. H.** | *Synopsis of Surgery* 10th Ed. | 1933 |
| **Hall, P.** | *Ultra Violet Rays* 4th Ed. | 1929 |
| **Ham, B. B.** | *Handbook of Sanitary Law* 11th Ed. | 1931 |
| **Hare, D. C.** | *Simple Instructions for Diabetic Patients* | 1933 |
| **Harington, C. R.** | *Thyroid Gland* | 1933 |
| **Harris, R. W., and Sack, L. S.** | *Medical Insurance Practice.* 3rd Ed. | 1929 |
| **Herelle, F. D'** | *Bactériophage et son Comportement* | 1926 |
| **Herries, N. E.** | *Guide to Birth Control Literature* | 1931 |
| **Hindley-Smith, T. D.** | *Chronic Rheumatism and the Pre-Rheumatic State* | 1932 |
| **Hobhouse, N.** | *Nervous Disorders in Infancy and Childhood* | 1932 |
| **Hunt, T. C.** | *Common Causes of Chronic Indigestion* | 1933 |
| **James, R. R.** | *Studies in the History of Ophthalmology in England prior to* 1800 | 1933 |
| **Jones, H. E.** | *Practical Points in Eye Surgery and Dressing* | 1933 |
| **Kemble, J.** | *Idols and Invalids* | [1933] |

| | | |
|---|---|---|
| **Kidd,** . .. .. .. | *Common Infections of the Female Urethra and Cervix* .. .. .. .. .. .. .. | 1929 |
| **M'Cabe, J.** .. .. | *Observations on the Cheltenham Waters* .. | 1820 |
| **McConnel, J. K.** .. | *Adjustment of Muscular Habits* .. .. .. | 1933 |
| **Martin, C. R. A.** .. | *Practical Food Inspection* Vol. II. .. .. | 1933 |
| **Massie, G.** .. .. .. | *Surgical Anatomy* .. .. .. .. .. .. .. | 1928 |
| **Ministry of Health** .. | *Final Report of Departmental Commission on Maternal Mortality and Morbidity* .. .. | 1932 |
| **Morison, J.** .. .. | *Bacteriophage in Treatment and Prevention of Disease* .. .. .. .. .. .. .. .. .. | 1932 |
| *Mouth Infections and their Relation to Systemic Disease.* Vol. 2 .. (8) | | 1933 |
| **Mozo, F. G.** .. .. | *Fisiopatologia del Metabolismo basal* .. (5) | |
| **Muir, R.** .. .. .. | *Textbook of Pathology* .. .. 3rd Ed. | 1933 |
| *Norske Medicinske Selskab.* 1833-1933. *Festskrift* .. .. .. .. | | 1933 |
| **Pasteur** (*Oeuvres de*) .. | *Tomes V et VI* .. .. .. .. .. .. | 1928-1933 |
| **Power, D'Arcy** .. .. | *Short History of Surgery* .. .. .. .. | 1933 |
| **Read, G. D.** .. .. | *Natural Childbirth* .. .. .. .. .. .. | 1933 |
| *Schola Medicinae Bristol.* 1833-1933 .. .. .. .. .. .. .. (13) | | 1933 |
| **Sewart, D.** .. .. | *Liver Diet Cookery Book* .. .. .. .. | 1933 |
| **Shattuck, G. C.** .. | *Peninsula of Yucatan* .. .. .. .. (3) | 1933 |
| **Smith, R. W. I.** .. | *English Speaking Students of Medicine at the University of Leyden* .. .. .. .. .. .. | 1932 |
| **Spira, L.** .. .. .. | *Clinical Aspect of Chronic Poisoning by Aluminium and its Alloys* .. .. .. | 1933 |
| **Stevens, W. M.** .. .. | *Resistance to Disease* .. .. .. .. .. .. | 1933 |
| *Treatise on Cold Mineral Waters* .. .. .. .. .. .. .. .. .. | | 1766 |
| **Walker, G. F.** .. .. | *Injured Workman* .. .. .. .. .. .. | 1933 |

TRANSACTIONS, REPORTS, JOURNALS, ETC.

| | |
|---|---|
| American Laryngological Association. Transactions of the 54th Annual Meeting .. .. .. .. .. .. .. .. .. .. .. .. (1) | 1932 |
| Boletin de la Universidad de Granada. Ano IV. No. 21 .. (4) | 1932 |
| Collected Papers of the Mayo Clinic and the Mayo Foundation. XXIV. | 1932 |
| College of Physicians of Philadelphia, Transactions. Vol. 54.. .. | 1932 |
| Medical Annual .. .. .. .. .. .. .. .. .. .. .. .. .. .. | 1933 |
| Medical Society's Transactions. Vol. VI. .. .. .. .. .. .. | 1933 |
| Otago University Medical School, Proceedings. No. 10 .. .. (7) | 1933 |
| Rockefeller Foundation, Annual Report .. .. .. .. .. .. (9) | 1932 |
| Royal College of Surgeons of England. Calendar .. .. .. (10) | 1933 |
| St. Thomas's Hospital Reports. Vol. LV. .. .. .. .. .. .. .. | 1931 |
| Surgical Clinics of North America. Vol. 13. Nos. 2, 3 and 4 .. | 1933 |
| Wilmer Ophthalmological Institute, Collected Reprints. Vol. 111 (12) | 1933 |

### Publications Received

From The Actinic Press :

*Therapeutic Uses of Infra-Red Rays.* 2nd Edition. By W. A. Troup, M.C., M.B., Ch.B. Price 6s. 6d.

From John Bale, Sons and Danielsson, Ltd. :

*Short History of Ophthalmology.* By Arnold Sorsby, M.D. Price 3s. 6d.

*Practical Points in Eye Surgery and Dressing.* By Hugh E. Jones, M.R.C.S., L.R.C.P. Price 2s. 6d.

From William Heinemann, Ltd. :

*Sex Efficiency Through Exercise.* By Th. H. Van de Velde, M.D. Price 25s.

*Clinical Contraception.* By Gladys M. Cox, M.B., B.S. Price 7s. 6d.

*Lettsom : His Life, Times, Friends and Descendants.* By J. J. Abraham. Price 30s.

*An Introduction to the Study of the Nervous System.* By E. E. Hewer, D.Sc., and G. M. Sandes, M.B., B.S. Price 21s.

From Jackson, Wylie & Co. :

*Physician as Man of Letters, Science and Action.* By T. K. Monro, M.A., M.D. Price 10s. 6d.

From H. K. Lewis & Co. Ltd. :

*The Adrenal Cortex.* By L. R. Broster, M.A., D.M., M.Ch., and H. W. C. Vines, M.A., M.D. Price 6s.

*Clinical Examination of the Nervous System.* 6th Edition. By G. H. Monrad-Krohn, M.D. Price 7s. 6d.

*Recent Progress in Medicine and Surgery.* 1919–1933. Edited by Sir John Collie, M.D. Price 10s.

*Treatment of Rheumatoid Arthritis and Sciatica.* By A. H. Douthwaite, M.D. Price 6s.

*Vaccine Therapy.* By H. T. Gillett, M.D. Price 5s.

From E. and S. Livingstone :

*Maternal Mortality and Morbidity.* By J. M. Munro Kerr, M.D. Price 25s.

From Longmans, Green & Co. :

*The Life of Edward Jenner.* 2nd Edition. By F. D. Drewitt. Price 6s.

From Macmillan & Co. :

*Stoke Park Monographs on Mental Deficiency and other Problems of the Human Brain and Mind.* No. 1. Edited by R. J. A. Berry, M.D. Price 10s. 6d.

From OLIVER AND BOYD :

*Sick Children.* 5th Edition. By JOHN THOMSON, M.D., LL.D., and LEONARD FINDLAY, M.D., D.Sc. Price 30s.

From OXFORD UNIVERSITY PRESS :

*Bone Growth in Health and Disease.* By H. A. HARRIS, D.Sc., M.B., B.S., B.Sc., M.R.C.S., M.R.C.P. Price 34s.

*The Enlarged Prostate and Prostatic Obstruction.* By KENNETH M. WALKER, M.A., M.B., B.C. Price 14s.

*Diseases of Infancy and Childhood.* 2 Vols. Edited by L. G. PARSONS, M.D., and S. BARLING, C.M.G. Price £4 4s. 0d.

*The Thyroid Gland.* By C. R. HARINGTON, M.A., Ph.D., F.R.S. Price 15s.

*Diseases of the Nervous System.* By W. RUSSELL BRAIN. Price 27s. 6d.

*Paralysis in Children.* By R. G. GORDON, M.D., D.Sc., and M. FORRESTER BROWN, M.D., M.S. Price 15s.

*Red Blood Cell Diameters.* By CECIL PRICE-JONES, M.B. Price 10s. 6d.

*Textbook of the Practice of Medicine.* 4th Edition. By F. W. PRICE, M.D. Price 36s.

*Diseases of the Eye.* By M. S. MAYOU. 4th Edition. Price 6s.

*Students' Guide to Fundus Appearances.* By FREDERICK RIDLEY and ARNOLD SORSBY. Price 2s. 6d.

*The Physician's Art.* By A. G. GIBSON. Price 7s. 6d.

From THE SOLLUX PUBLISHING CO. :

*Actinotherapy Technique.* 1933. Price 6s.

From JOHN WRIGHT & SONS, LTD. :

*British Journal of Surgery.* Vol. XXI. Nos. 81, 82. 1933. Price 12s. 6d. each.

*Baths and Medicinal Waters of Britain and Europe.* By M. G. FOSTER, M.A., M.D. Price 12s. 6d.

*An Outline of Surgery for Nurses.* By D. W. DANIELS, M.D. Price 2s. 6d.

*Blood Pictures.* 3rd Edition. By C. PRICE-JONES, M.B. Price 6s. 6d.

*Some Thoughts on Asthma.* By A. J. D. CAMERON. Price 7s. 6d.

*Elementary Bandaging and Surgical Dressing.* By WALTER PYE. Price 3s. 6d.

*Chronic Nasal Sinusitis.* 2nd Edition. By P. WATSON-WILLIAMS. Price 15s.

*British Journal of Surgery.* General index to Vols. XI.—XX. Price 10s. 6d.

*Synopsis of Forensic Medicine and Toxicology.* By E. W. C. THOMAS, M.D., B.Sc., D.P.H. Price 7s. 6d.

*Miner's Nystagmus.* By F. O'SULLIVAN, M.B., Ch.B., B.A.O. Price 5s.

### Local Medical Notes

EXAMINATION RESULTS.

**University of Bristol.**—Students of the University have recently passed the following examinations:—

M.B., Ch.B.—*First Examination.*—P. A. Bennett, K. J. V. Carlson, A. A. K. Datta, M. Dworkis, W. H. G. Elliott, M. M. Lewis, Joan E. Mackworth, G. R. E. Maxted, J. S. Richardson, E. W. J. Townsend. *Physics:* Kathleen Byrt. *Chemistry:* Joyce S. Miller, K. Savill.

M.B., Ch.B.—*Supplementary First Examination.*—K. A. Hall, K. Savill.

M.B., Ch.B. — *Second Examination. Part I. Organic Chemistry:* K. J. V. Carlson, R. L. J. Derham, P. K. Jenkins.

M.B., Ch.B.—*Final Examination. Part I. (including Forensic Medicine and Toxicology):* V. T. Baxter (with distinction in Forensic Medicine and Toxicology), Kathleen G. Brimelow (with distinction in Forensic Medicine and Toxicology), F. C. Collingwood, Theodora M. Crabtree, J. G. Field, P. N. Heron, A. E. Jowett, S. D. Loxton (with distinction in Materia Medica and Pharmacy, Pharmacology and Therapeutics), A. W. Woolley (with distinction in Forensic Medicine and Toxicology).

M.B., Ch.B.—*Final Examination. Part II.:* S. B. Adams. *Group II.:* Frances E. Powell.

B.D.S.—*First Examination. Chemistry:* F. P. Ewens. P. H. S. Paine. *Physics:* J. C. Jowett.

B.D.S.—*Supplementary First Examination:* F. P. Ewens.

B.D.S.—*Second Examination. Anatomy and Physiology :* C. A. Blanden, C. C. B. Plumley.

L.D.S. — *Preliminary Science Examination. Biology :* H. W. Williams, M. J. Banbury, C. C. Moore.

L.D.S. — *Supplementary Preliminary Science. Physics and Chemistry :* J. F. Durie, C. C. Moore. *Biology :* J. B. Inverdale.

L.D.S.—*First Professional Examination. Anatomy and Physiology :* M. J. Banbury.

L.D.S. — *Second Professional Examination. Dental Mechanics and Dental Metallurgy :* B. E. Ffrench, Frances M. Orton, L. E. Scull, R. E. Buchan, D. E. Royal. *Dental Materia Medica :* J. G. Clancy.

**Conjoint Board.**—M.R.C.S., L.R.C.P.—*Chemistry :* R. T. Moore, A. C. F. Alexander. *Physics :* R. T. Moore. *Pharmacology and Materia Medica :* T. H. Taylor. *Pathology :* A. G. W. Branch, R. H. Moodie, J. N. Heales, R. A. Mathews, Dorothy E. Barber, Rosalind M. S. Derham. A. V. House.* *Medicine :* A. V. House,* W. R. Cambridge,* Winifred M. Hill,* E. O. Walker.* *Surgery :* D. A. J. Hunwick,* Gwladys R. Llewelyn, A. V. House,* W. R. Cambridge.* *Midwifery :* J. N. Heales, Dorothy E. Barber, Rosalind M. S. Derham, C. H. G. Price, Irene G. Hamilton. *Anatomy and Physiology :* E. J. Lace. *Physiology :* J. D. Buckner.

L.D.S., R.C.S.—*Final Examination. First Professional Examination. Part I. Dental Mechanics :* D. J. Dymond, W. White, *Part II. Dental Metallurgy :* J. T. S. Hutchins. *Part III. Anatomy and Physiology :* J. G. Jones.

L.D.S., R.C.S.—*Final Examination. Part I. :* T. H. Williams, G. L. R. Welch. *Part II. :* A. C. Davies,* D. Gent,* N. G. Harrison,* N. E. Miles,* H. F. Vere,* O. F. Harley, J. D. Rees.

* Qualified.

**Society of Apothecaries of London.**—L.S.A.—*Final Examination. Medicine and Midwifery:* G. C. Canaval.* *Surgery and Forensic Medicine:* G. C. Canaval.* *Medicine:* Helene L. Canaval.

* Qualified.

---

### FIFTY-YEAR INDEX TO THE "JOURNAL."

It has, unfortunately, proved impossible to complete this in time for inclusion with the present issue. It will be sent to subscribers as soon as it is ready.

### Index

Appointments—
Brevet-Col. H. E. Stanger-Leathes.—Honorary Surgeon to H.M. the King, 71.
V. B. Green-Armytage.—Gynæcologist to the West London Hospital, 71.
J. E. Newton.—Casualty Officer at West London Hospital, 71.
J. R. Charles.—Consulting Physician to Bristol Royal Infirmary, 71.
W. K. Wills.—Consulting Physician to Skin Department of Bristol General Hospital, 71.
N. F. C. Burgess.—Physician to Skin Department of Bristol General Hospital, 71.
H. H. Carleton.—Physician to Bristol General Hospital, 71.
C. B. Perry.—Assistant Physician to Bristol General Hospital, 71.

Bacillus coli infection of the female urinary tract.—H. J. D. Smythe, 243.
Bath and Bristol Surgical Club—
See under Societies.
Blood - pressure, Need for standard method of estimating the.—G. A. Stephens, 121.
Bristol Medical School—
Centenary of the foundation of, 183.
Centenary, Programme of celebrations, 134.
History of, by G. Parker (Review), 199.
*Bristol Medico - Chirurgical Journal*, History of.—P. Watson-Williams, 151.
Bristol Medico-Chirurgical Society—
See under Societies.
Bristol University, Opening of the Institute of Preventive Medicine, 267.
Bristol's contributions to medical progress, 165.
Burden Mental Research Trust, 58.

Cancer, Recent advances in the ætiology, diagnosis and treatment of.—C. A. Joll, 201.
Carey Coombs Memorial, 134.
Carling, E. R.—Surgery for pain, 81.
Chitty, H.—Anterior poliomyelitis, or infantile paralysis, 37.
Cholera in Bristol, J. A. Symonds and, 57.
Coombs, C. F.—Thirty years' progress in the study of rheumatic heart disease, 93.

Female urinary tract, Bacillus coli infection of.—H. J. D. Smythe, 243.

Groves, E. W. H.—A surgical adventure : an autobiographical sketch, 1.

Heart disease, rheumatic, Thirty years' progress in the study of.—C. F. Coombs, 93.

Infantile paralysis, or anterior poliomyelitis.—H. Chitty, 37.
Institute of Preventive Medicine, University of Bristol, 267.

Joll, C. A.—Recent advances in the ætiology, diagnosis and treatment of cancer, 201.

Library Accessions, 66, 142, 297.
Long Fox Memorial Lecture, 1933.—C. A. Joll.—Recent advances in the ætiology, diagnosis and treatment of cancer, 201.

Mucous membranes, Familial multiple telangiectases of the skin and.—G. R. Scarff, 113.

Obituaries—
Evelyn R. Bates, 63.
C. A. Calmette, 287.
D. S. Davies, 135.
C. Elliott, 62.
S. J. H. Griffiths, 62.
W. H. Harsant, 60.
Sir P. Sargent, 59.
W. A. Smith, 286.

Paralysis, infantile, or anterior poliomyelitis.—H. Chitty, 37.
Paul Bush Gold Medal, 1933.—A. A. G. Flemming, 71.
Poliomyelitis, anterior, or infantile paralysis.—H. Chitty, 37.
Pridie, K. H.—The ventral position in the treatment of tuberculous spine, 261.
Prostate, enlarged.—A. R. Short, 23.

Radium therapy, The present position of.—S. B. Wigoder, 233.
Rheumatic heart disease, Thirty years' progress in the study of.—C. F. Coombs, 93.
Royal Society of Medicine (Section of Surgery)—
See under Societies.

Scarff, G. R. —Familial multiple telangiectases of the skin and mucous membranes, 113.
Short, A. R.—Enlarged prostate, 23.
Skin and mucous membranes, Familial multiple telangiectases of.—G. R. Scarff, 113.
Smythe, H. J. D.—Bacillus coli infection of the female urinary tract, 243.
Societies—
Bristol Medico-Chirurgical Society.
Pseudo - hypertrophic paralysis treated by sodium fluoride.—A. T. Todd (2 Cases), 65.
Treatment of asthma.—A. T. Todd and J. A. James, 65.
Remarks by
E. W. H. Groves.
J. O. Symes.
H. H. Carleton.
C. B. Perry.
C. A. H. Gee.
A. J. M. Wright.
J. A. Birrell.
F. H. Bodman.
F. J. Hector.
J. A. Nixon.
Surgery for pain.—E. R. Carling, 65.
Oesophageal obstruction (2 Cases).—E. Watson-Williams, 138.
Remarks by A. J. M. Wright.
B Coli infections of the female urinary tract.—H. J. D. Smythe, 138.
Remarks by
E. W. H. Groves.
R. S. S. Statham.
F. J. Hector.
F. H. Bodman.
C. F. Walters.
Common nervous disorders in general practice.—F. H. Bodman, 139.
Remarks by
E. W. H. Groves.
H. H. Carleton.
E. Casson.
J. R. Charles.
I. B. Saxby.
J. A. Nixon.
A. T. Tódd.
R. R. Garden.
R. G. de M. Rudolph.
Gastric surgery.—A. W. Adams, 139.
Remarks by
E. W. H. Groves.
R. C. Clarke.
E. Watson-Williams.
C. A. Moore.
H. Chitty.
Early results of radium treatment in operable carcinoma.—S. B. Wigoder, 140.
Remarks by
E. W. H. Groves.
M. F. Forrester Brown.
D. Wood.
C. A. Moore.
E. Watson-Williams.
Bath and Bristol Surgical Club, 140.
Royal Society of Medicine (Section of Surgery). Visit to Bristol, 140.
Spine, tuberculous, Ventral position in treatment of.—K. H. Pridie, 261.
Stephens, G. A.—Need for a standard method of estimating blood-pressure, 121.
Symonds, J. A., and cholera in Bristol, 57.

Telangiectases of the skin and mucous membranes, familial multiple.—G. R. Scarff, 113.
Tuberculous spine, Ventral position in treatment of.—K. H. Pridie, 261.

Urinary tract, Female, Bacillus coli infection of.—H. J. D. Smythe, 243.

Ventral position in the treatment of tuberculous spine.—K. H. Pridie, 261.

Watson-Williams, P.—History of *The Bristol Medico-Chirurgical Journal*, 151.
Wigoder, S. B.—The present position of radium therapy, 233.

